# Age and sex effects on paired-pulse suppression and prepulse inhibition of auditory evoked potentials

**DOI:** 10.3389/fnins.2024.1378619

**Published:** 2024-04-09

**Authors:** Koji Inui, Nobuyuki Takeuchi, Bayasgalan Borgil, Megumi Shingaki, Shunsuke Sugiyama, Tomoya Taniguchi, Makoto Nishihara, Takayasu Watanabe, Dai Suzuki, Eishi Motomura, Tetsuo Kida

**Affiliations:** ^1^Department of Functioning and Disability, Institute for Developmental Research, Aichi Developmental Disability Center, Kasugai, Japan; ^2^Section of Brain Function Information, National Institute for Physiological Sciences, Okazaki, Japan; ^3^Department of Psychiatry, Okazaki City Hospital, Okazaki, Japan; ^4^Department of Psychiatry, Gifu University Graduate School of Medicine, Gifu, Japan; ^5^Department of Anesthesiology, Nagoya University Graduate School of Medicine, Nagoya, Japan; ^6^Multidisciplinary Pain Center, Aichi Medical University, Nagakute, Japan; ^7^Department of Clinical Laboratory, Mie University Hospital, Tsu, Japan; ^8^Department of Neuropsychiatry, Mie University Graduate School of Medicine, Tsu, Japan

**Keywords:** aging, change-related potential, P50 gating, sensory suppression, sensory gating

## Abstract

Responses to a sensory stimulus are inhibited by a preceding stimulus; if the two stimuli are identical, paired-pulse suppression (PPS) occurs; if the preceding stimulus is too weak to reliably elicit the target response, prepulse inhibition (PPI) occurs. PPS and PPI represent excitability changes in neural circuits induced by the first stimulus, but involve different mechanisms and are impaired in different diseases, e.g., impaired PPS in schizophrenia and Alzheimer’s disease and impaired PPI in schizophrenia and movement disorders. Therefore, these measures provide information on several inhibitory mechanisms that may have roles in clinical conditions. In the present study, PPS and PPI of the auditory change-related cortical response were examined to establish normative data on healthy subjects (35 females and 32 males, aged 19–70 years). We also investigated the effects of age and sex on PPS and PPI to clarify whether these variables need to be considered as biases. The test response was elicited by an abrupt increase in sound pressure in a continuous sound and was recorded by electroencephalography. In the PPS experiment, the two change stimuli to elicit the cortical response were a 15-dB increase from the background of 65 dB separated by 600 ms. In the PPI experiment, the prepulse and test stimuli were 2- and 10-dB increases, respectively, with an interval of 50 ms. The results obtained showed that sex exerted similar effects on the two measures, with females having stronger test responses and weaker inhibition. On the other hand, age exerted different effects: aging correlated with stronger test responses and weaker inhibition in the PPS experiment, but had no effects in the PPI experiment. The present results suggest age and sex biases in addition to normative data on PPS and PPI of auditory change-related potentials. PPS and PPI, as well as other similar paradigms, such as P50 gating, may have different and common mechanisms. Collectively, they may provide insights into the pathophysiologies of diseases with impaired inhibitory function.

## 1 Introduction

Paired-pulse suppression (PPS) describes short-term plasticity in neural circuits in which the response to a stimulus is inhibited by the preceding same stimulus. The activity-dependent modulation of a circuit is a primary form of synaptic modulation and is important for generating appropriate outputs with various functional roles (O’Donovan and Rinzel, 1997). In humans, PPS is typically evaluated using the P50 gating paradigm, in which two identical sound stimuli are presented 500 ms apart and the degree of inhibition is assessed using the P50 component of auditory evoked potentials (AEPs) elicited by the two stimuli (Adler et al., 1982); the PPS value indicates how strongly the first stimulus filters out the response to the second stimulus. The PPS of P50 gating is impaired in several diseases, including schizophrenia (Adler et al., 1982), bipolar disorder (Lijffijt et al., 2009b), and Alzheimer’s disease (Jessen et al., 2001). In another paradigm for assessing sensory inhibition, the first stimulus is weak enough to elicit reliable responses, but inhibits subsequent responses to a strong sensory stimulus. This is called prepulse inhibition (PPI) (Graham, 1975). Startle reflexes are generally elicited by a loud sound and the blink reflex in humans is recorded from the orbitalis muscle using electromyography with an electrode placed on the lower eyelid. When a weak sound, or prepulse, precedes the reflex-evoking test stimulus, the magnitude of the startle reflex is reduced. PPI deficits are widely known in neuropsychiatric diseases, such as schizophrenia (Braff, 2010), movement disorders (Swerdlow, 2013), and developmental disorders (McAlonan et al., 2002). Previous animal studies revealed some neural circuits of the acoustic startle reflex in the brainstem, which did not involve cortical regions (Lee et al., 1996). Similar to PPS, PPI is considered to represent a gating mechanism; however, previous studies showed that these two measures reflected different mechanisms (Light and Braff, 2001; Brenner et al., 2004; Braff et al., 2007).

Several neural mechanisms were shown to be involved in PPS in animal studies, e.g., a release-independent presynaptic mechanism and release-dependent postsynaptic mechanism (Kirischuk et al., 2002). Therefore, impairments in electrophysiological measures of inhibition in some diseases may reflect abnormalities in different mechanisms. For example, reduced P50 gating in patients with schizophrenia and Alzheimer’s disease may reflect different aspects of the measure. On the other hand, impairments in different diseases may also represent common mechanisms (Swerdlow, 2013). PPI of different methods may involve distinct mechanisms. Since the mechanisms underlying sensory inhibition remain unclear, information from multiple sources needs to be accumulated.

We previously examined a similar phenomenon using auditory change-related cortical responses. A change-related cortical response is elicited by abrupt changes in sensory inputs and recorded using electroencephalography and magnetoencephalography (Inui et al., 2010a,b). It represents automatic brain processes to detect salient sensory events and is common across auditory (Yamashiro et al., 2009a, 2011; Nishihara et al., 2011), somatosensory (Yamashiro et al., 2009b; Otsuru et al., 2011), and visual (Tanaka et al., 2009a,b; Urakawa et al., 2010a,b) systems. When the same change stimulus is presented twice 600 ms apart, a similar change-related response is elicited twice, with the second response being smaller, i.e., PPS (Takeuchi et al., 2017, 2018). If a weak change stimulus is presented 10–800 ms before the test change stimulus, the magnitude of the test response is smaller than when the test stimulus is presented alone, i.e., PPI (Inui et al., 2012, 2016). Although PPI is generally used for the startle reflex, this phenomenon indicates that a weak preceding input affects excitability somewhere in its own pathway. Therefore, similar mechanisms may exist in other neural systems. In the present study, a reduction in the test response by a weak leading stimulus, irrespective of the neural circuit, is referred to as PPI. In addition to the startle reflex and cortical responses, we showed that the R1 component of the trigeminal blink reflex with an oligosynaptic pathway showed PPI over a similar time course to conventional PPI (Inui et al., 2023). In the present study, the auditory change-related response was used as the test response. Previous studies on PPI and PPS reported high test–retest reliability when the change-related cortical response was used (Takeuchi et al., 2021a,b).

One of the issues associated with interpreting PPI and PPS data is that they are affected by age and sex, similar to many other indexes. Previous studies using P50 gating and PPI of the startle reflex revealed significant sex and age effects (Swerdlow et al., 1993; Kumari et al., 2003; Fuerst et al., 2007; de Oliveira et al., 2023). Accumulating evidence has shown that age and sex widely affect brain function, and these variables may have an impact on the findings or even conclusion of a study (Cahill, 2012). Since these effects on PPI or PPS of the change-related response have not been investigated, the present study was undertaken. These effects need to be confirmed before current methods are applied in clinical studies. Therefore, the present study was conducted to obtain normative data on PPI and PPS as indexed by the change-related cortical response and to establish whether age and sex need to be considered as bias factors.

## 2 Methods

The present study was approved in advance by the Ethics Committee of the Aichi Developmental Disability Center, Kasugai, Japan (approval number: RC0502) and conducted in accordance with the Declaration of Helsinki. Written consent was obtained from all subjects. The experiment was performed on 67 healthy non-smokers (35 females and 32 males) aged 36.8 ± 12.3 (range, 19–70) years. None of the subjects were treated for neurological or mental diseases or substance abuse in the last 2 years. They had normal hearing as assessed by an audiometer (AA-71, Rion, Tokyo, Japan). Two experiments were conducted on all subjects, Experiment 1 (Exp1) and Experiment 2 (Exp2), in that order. The former was the PPS experiment, while the latter was the PPI experiment. Although females are always tested in the luteal phase of the menstrual cycle in standard studies, information on menstruation was not obtained in the present study.

### 2.1 Auditory stimuli

**Figure 1:**
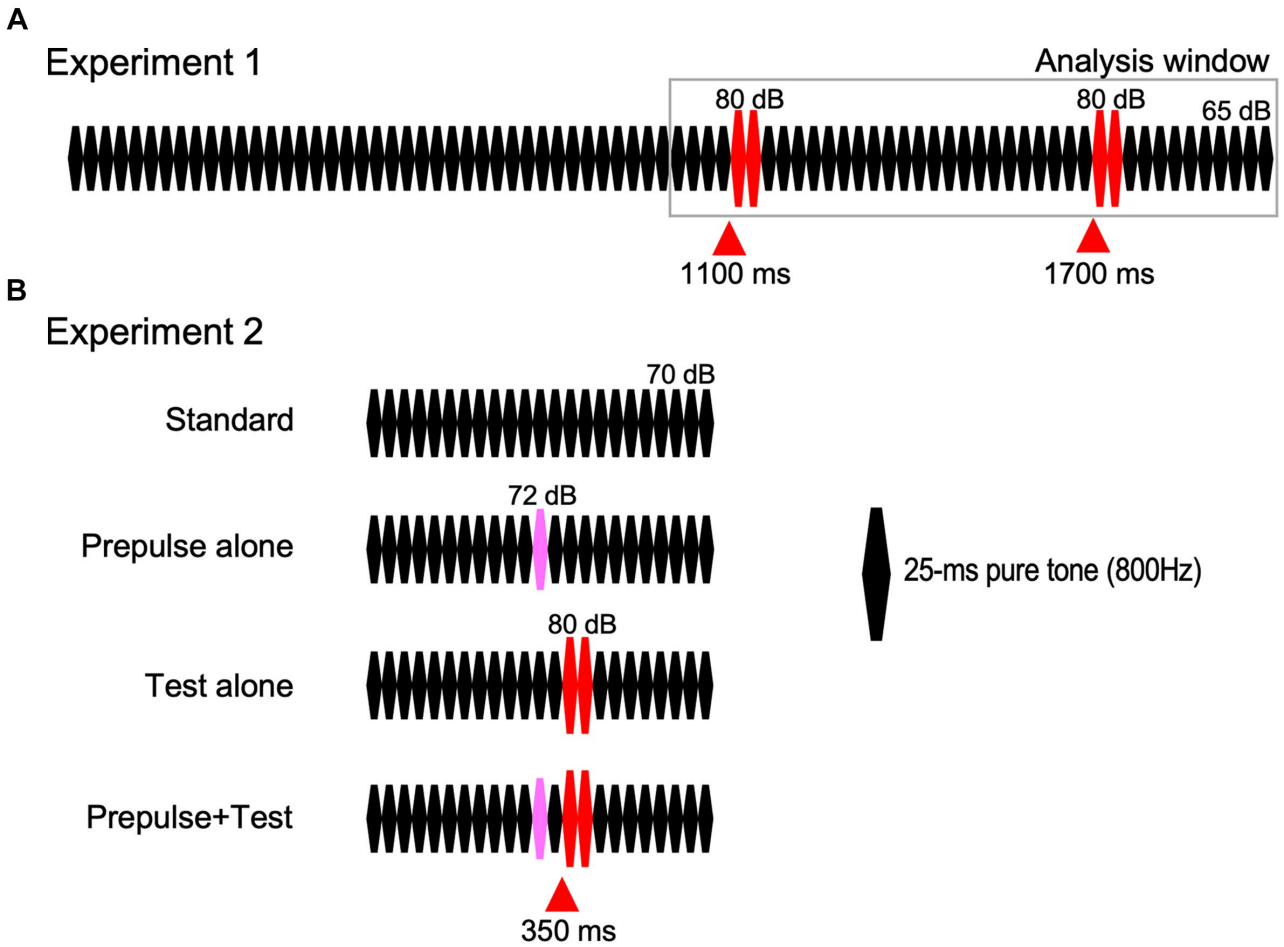
Sound stimuli. Red-filled triangles indicate the timing of the abrupt sound pressure increase for eliciting the test response.

Repeats of 25-ms pure tones (800 Hz, rise and fall times of 5 ms) were used for auditory stimuli because abrupt changes in any sound feature are possible without any undesired effect (Inui et al., 2010a). Although the carrier may be any audible frequency, we have used 800 Hz because it has the advantage of varying the sound location by manipulating the interaural time difference (Inui et al., 2010a; Akiyama et al., 2011; Ohoyama et al., 2012). The standard sound stimulus was 80 repeats of the 25-ms tone at 65 dB SPL (Figure 1A) in Exp1 and 24 repeats at 70 dB in Exp2. The change stimulus to evoke the change-related cortical response was two consecutive 25-ms tones of 80 dB (Takeuchi et al., 2017). In Exp1, two identical change stimuli were inserted at 1,100 and 1,700 ms of the 2000-ms standard stimulus. Therefore, sound pressure was abruptly increased twice with a 600-ms interval. In Exp2, there were four types of sound stimuli (Figure 1B): standard sound without any changes, a test alone stimulus (Test alone) with a sound pressure increase to 80 dB at 350 ms, a prepulse alone stimulus with a slight increase in sound pressure of 2 dB at 300 ms, and a test + prepulse stimulus with the prepulse and test. Therefore, the prepulse–test interval was 50 ms or the inter-stimulus interval was 25 ms. Sound stimuli were created using a PC (Windows XP, 32 bit) and delivered through a headphone.

### 2.2 Recordings

Auditory evoked potentials were recorded using an EMG/EP measuring system (MEB-2300, Nihon Kohden, Tokyo) at a sampling rate of 1,000 Hz. An exploring electrode was placed at FzCz referenced to the linked P9-P10 (Inui et al., 2010a). The activity at FzCz was the sum of both hemispheres. To reject trials with eye blinks, a pair of electrodes were placed on the supra- and infra-orbit of the right eye. Impedance for all the electrodes was <5 kΩ. The analysis window was 1,000 to 2,000 ms from the onset of the auditory stimulus in Exp1 (Figure 1A) and from 100 ms before to 600 ms after the stimulus onset in Exp2. The analog filter was 0.5–100 Hz. Trials with activity >100 μV were automatically rejected from averaging.

### 2.3 Procedures

Subjects were seated on a chair and watched a silent movie on a screen 1.5 m in front of them throughout the recording. They were instructed to ignore stimuli and concentrate on the movie (Inui et al., 2010a). Exp1 was conducted before Exp2. In Exp1, the sound stimulus was presented with a trial-trial interval of 2,200 ms or inter-trial interval of 200 ms. After completing an average of 120 trials, Exp2 was immediately initiated. Four sound stimuli were presented randomly at a trial-trial interval of 800 ms or inter-trial interval of 200 ms. A total of 120 artifact-free epochs were averaged for each sound stimulus. Order effects were unlikely because the change-related cortical response is resistant to repetition (Inui et al., 2010a).

### 2.4 Analysis

**Figure 2:**
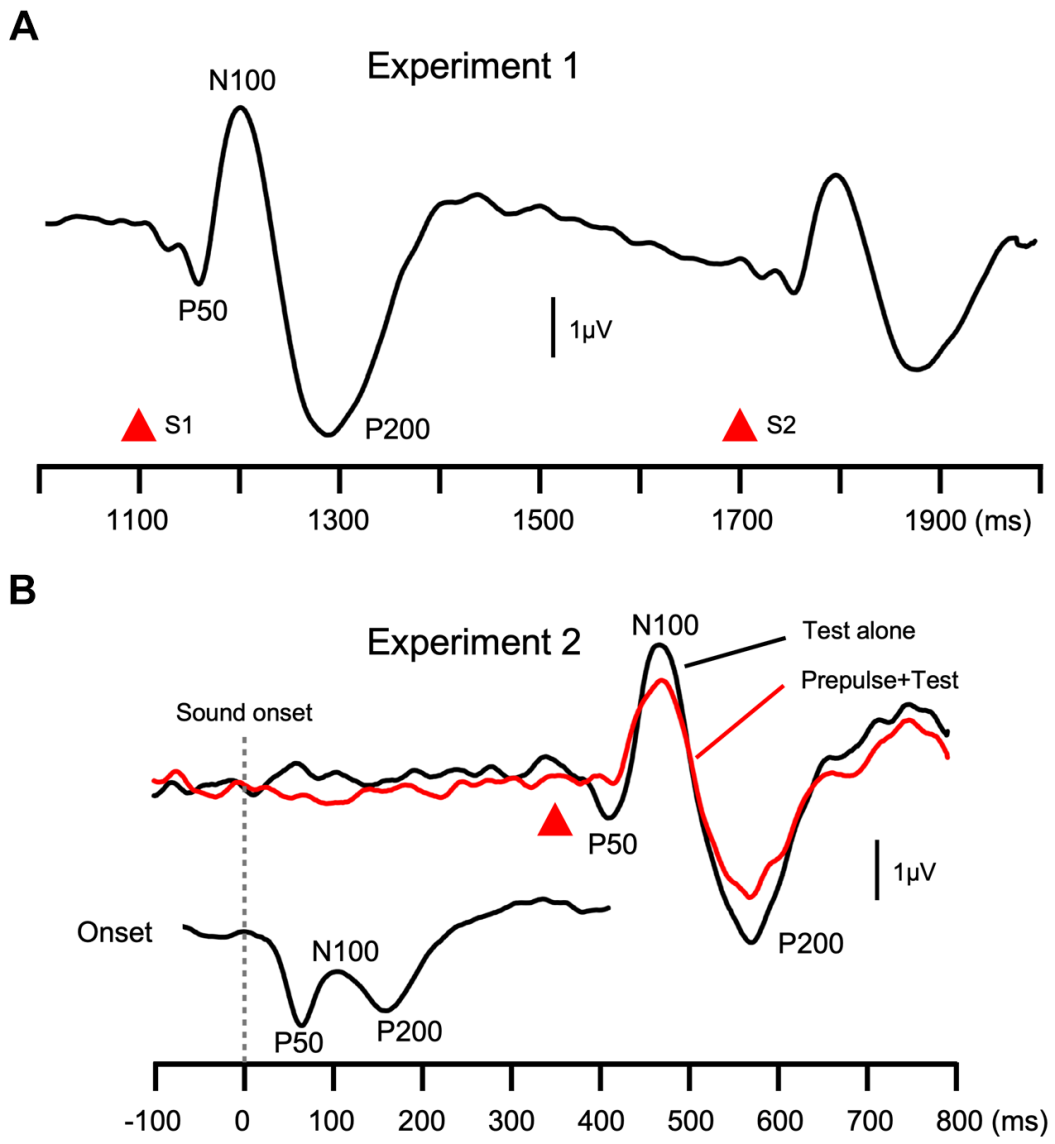
Grand-averaged waveforms. Grand-averaged waveforms across all subjects in Experiment 1 **(A)** and 2 **(B)** are shown. The peak-to-peak-to-peak amplitude (P50-N100-P200) was used as the magnitude of the test response.

Recorded signals were subjected to band-pass filtering of 0.9–35 Hz (Takeuchi et al., 2023). Any abrupt changes in a continuous sound elicit triphasic change-related cortical responses with peaks at approximately at 60, 120, and 200 ms (P50/N100/P200, Figure 2) (Inui et al., 2010a). In the present study, the peak-to-peak-to-peak amplitude was used to evaluate the magnitude of the response. This procedure minimizes issues associated with a baseline shift (Inui et al., 2010b). The P50-N100-P200 amplitude was calculated using the following formula: (P50-N100 peak-to-peak amplitude + N100-P200 peak-to-peak amplitude)/2.

In Exp1, there was an abrupt change in sound pressure twice, S1 and S2, which elicited S1-evoked change-related responses and smaller S2-related responses. The degree of inhibition of the S2 response (PPS) was calculated using the following formula: (S1 amplitude – S2 amplitude)/S1 amplitude × 100. In Exp2, subtraction procedures were necessary to calculate the response amplitude (Inui et al., 2010a). To obtain the test alone response, responses to the standard were subtracted from those to the test alone stimulus. Prepulse + Test responses were obtained by subtracting responses to the prepulse alone stimulus from those to the test + prepulse stimulus. The amplitude of the response was measured using difference waveforms. The degree of PPI was calculated as follows: (Test alone amplitude – Prepulse + Test amplitude)/Test alone amplitude × 100.

In addition to the test response and its inhibition, the amplitude of the onset response was analyzed in Exp2. The onset response was obtained by averaging all trials. Similar to the change-related response, the peak-to-peak-to-peak amplitude was measured (Figure 2B). Therefore, five electrophysiological variables were used in the present study: the amplitude of the response to the first stimulus (S1) and the degree of inhibition of the second stimulus (PPS) in Exp1 and the amplitude of the Test alone response (Test Alone), PPI, and the amplitude of the onset response (Onset) in Exp2.

The effects of sex on these five variables were assessed using a discriminant analysis. To assess the contributions of each variable to separate sexes, the standardized canonical discriminant function coefficient was calculated. The relationships between age and variables were evaluated using a partial correlation analysis controlling for sex in addition to Pearson’s correlation coefficients. The significance of differences was set at 0.05. All statistical analyses were performed using SPSS ver. 24.

## 3 Results

### 3.1 Effects of sex and age

Females (39.1 ± 12.6 y.o.) were slightly older than males (34.0 ± 11.4) (*p* = 0.09). In both experiments, the abrupt change in sound pressure (Figure 1) elicited triphasic change-related cortical responses with peaks at approximately 60, 120, and 200 ms (P50/N100/P200), consistent with previous findings (Inui et al., 2010a). Grand-averaged waveforms across subjects are shown in Figure 2. Conditioning stimuli significantly reduced the amplitude of subsequent test responses in both Exp1 (paired *t*-test, *p* = 1.3 × 10^−20^) and Exp2 (2.0 × 10^−11^). The effects of sex were examined using a discriminant analysis with five electrophysiological measures as independent variables. Results showed that the function was significant (λ = 0.70, *p* = 3.9 × 10^−4^) with larger contributions of Onset, S1, Test Alone, and PPS in that order as judged by standardized canonical discriminant coefficients (Table 1): females had stronger Test Alone, Onset, and S1 and weaker PPS than males. The results of *t*-tests obtained using each evoked potential component (P50, N100, and P200) and peak-to-peak amplitudes are shown in Supplementary Table S1.

**Table 1:** Effects of sex.

	Female	Male	*r*-value^*^
S1 (μV)	6.5 (2.7)	5.5 (2.2)	0.50
PPS (%)	30.1 (18.0)	36.9 (11.6)	−0.23
Test alone (μV)	6.8 (2.5)	5.6 (2.2)	0.44
PPI (%)	22.8 (25.8)	25.2 (22.8)	−0.13
Onset (μV)	4.3 (1.5)	2.8 (1.1)	0.99

The results of a discriminant analysis are shown. Data are shown as means (SD). ^*^Standardized canonical discriminant function coefficient. S1, the amplitude of the first response in Experiment 1; PPS, paired-pulse suppression; PPI, prepulse inhibition.

**Figure 3:**
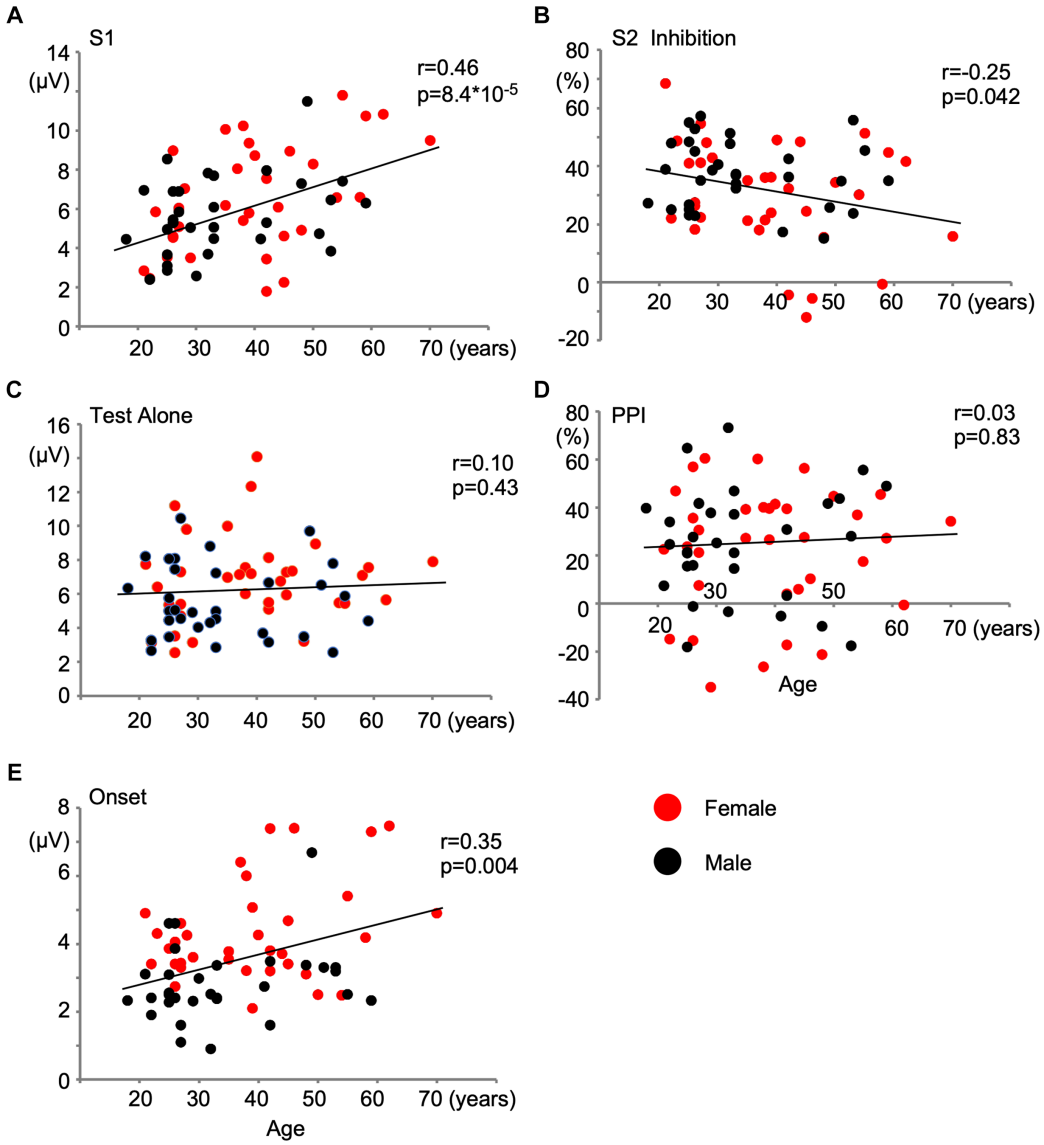
Effects of age on change-related cortical response and inhibition. Scatter plots show the relationship of age with the amplitude of the first response **(A)** and inhibition of the second response **(B)** in Experiment 1, and with the amplitude of the test alone response **(C)**, prepulse inhibition **(D)**, and the amplitude of the onset response **(E)** in Experiment 2. Correlation coefficients and *t*-values obtained by the simple linear regression analysis are shown.

The effects of age were assessed using a partial correlation analysis controlling for sex. As shown in Table 2, aging significantly affected S1 (*p* = 2.4 × 10^−4^) and Onset (*p* = 0.022). PPS showed a weak negative correlation with age (*r* = −0.21, *p* = 0.087). Older subjects showed stronger S1 and weaker PPS in Exp1 and stronger Onset in Exp2. On the other hand, the amplitude of the test alone response and its PPI in Exp2 were not affected by age. Figure 3 shows the relationships between age and five variables with regression lines obtained from the simple linear regression analysis. Results based on each evoked potential component are presented in Supplementary Table S2.

**Table 2:** Correlations between age and five variables.

		Simple^*^	Partial^**^
Age	S1	0.46	0.44
	PPS	−0.25	−0.21
	Test alone	0.10	0.05
	PPI	0.03	0.04
	Onset	0.35	0.28

^*^Simple linear correlation coefficients (Pearson’s). ^**^Partial correlation coefficients controlling for sex.

### 3.2 Relationships among variables

Some additional comparisons were performed among variables. Table 3 shows the results of partial correlation analyses with sex and age as covariates. Since the change stimulus was the same, the amplitudes of the test responses, S1 and Test Alone, correlated between the two experiments (*r* = 0.64). However, the degree of inhibition did not correlate between the two experiments (*r* = 0.08), which suggested that these two inhibitions were controlled by different mechanisms. As previously reported (Inui et al., 2012; Kodaira et al., 2013), a correlation was observed between Test Alone and PPI in Exp2: subjects with stronger Test alone responses showed stronger PPI (*r* = 0.44). However, this was not the case in Exp1: the S1 amplitude and PPS did not correlate (*r* = 0.09), which was investigated for the first time in the present study. A positive correlation was noted among S1, Test alone, and Onset.

**Table 3:** Correlations among five variables.

Age and sex covariates	S1	PPS	Test alone	PPI	Onset
S1	—				
PPS	0.09	—			
Test alone	0.64	0.03	—		
PPI	0.22	0.08	0.44	—	
Onset	0.52	−0.06	0.33	−0.03	—

Partial correlation coefficients while controlling for sex and age.

## 4 Discussion

The present study showed the significant effects of sex and age on electrophysiological measures. The amplitude of the test response in both experiments and that of the onset response were greater for females, while inhibition in both experiments was slightly weaker (Table 1). Aging correlated with the greater S1 amplitude and weaker PPS in Exp1 and the stronger Onset response in Exp2, but did not affect the amplitude of the Test alone response or its PPI (Table 2). In the brain, sex differences have been reported in cognitive functions (Delgado and Prieto, 1996), structures (de Courten-Myers, 1999), connectivity (Ingalhalikar et al., 2014), gene expression (Gegenhuber and Tollkuhn, 2020), neurotransmission (Pandya et al., 2019), and neurodegeneration (Cuenca-Bermejo et al., 2023). These differences may lead to sex differences in vulnerability to some diseases, such as autism spectrum disorder and mood disorder, clinical presentation, the severity of symptoms, the course of illness, and treatment outcomes (Vega et al., 2011; Fernández-Guasti et al., 2012; Fernando et al., 2020; Ziemka-Nalecz et al., 2023). Aging affects some cognitive functions, but not others (Rey-Mermet and Gade, 2018). Since the mechanisms underlying these effects in clinical aspects have not yet been elucidated in detail, the accumulation of information from multiple sources is needed. Therefore, the present study was performed to establish normative data and identify possible biases with a view to their use in clinical research.

### 4.1 Effects of sex

The results obtained herein revealed clear sex effects on some variables. Females showed weaker inhibition and stronger test responses than males. Although PPI of the cortical response differed from previously reported PPI in neural circuits, similarities and dissimilarities were examined between previous studies and the present investigation. Weaker inhibition in females was consistent with the findings of previous studies using PPI of the acoustic startle reflex (Swerdlow et al., 1993; Kumari et al., 2003; Aasen et al., 2005; Naysmith et al., 2022) and PPI of the trigeminal blink reflex (Kofler et al., 2013). Since the target responses and, hence, neural circuits examined in these studies differed from that of the present study, the similar results and findings reported are important because they suggest common mechanisms. One explanation for the mechanisms underlying sex differences is ovarian hormones. PPI of the startle reflex fluctuates with the menstrual cycle, with weaker PPI in the luteal phase (Swerdlow et al., 1997; Jovanovic et al., 2004). More specifically, a larger increase in progesterone levels in the luteal phase is associated with a smaller decrease in PPI from the follicular to luteal phase (Kumari et al., 2010). As a possible mechanism, the female hormone cycle affects the dopaminergic system (Zachry et al., 2021), which, in turn, has an impact on PPI (Zhang et al., 2000) such that an increase in the release of dopamine decreases PPI. Therefore, weaker inhibition in females in the present study may be attributed to young female subjects being in the luteal phase at the time of testing.

Another interesting effect of sex is that prepulse facilitation (PPF) was shown to be stronger in females in studies with the auditory startle reflex (Aasen et al., 2005). Weaker PPI and stronger PPF in females indicated that this bias is based on a general shift in facilitation/inhibition in the direction of facilitation in women relative to that in men (Kumari et al., 2003). In the present study, augmented S2 responses were only observed in females in Exp1 (Figure 3B). PPF was also slightly stronger in females in Exp2 (Figure 3D): PPF was present in 7 females with a mean value of −18.7% of PPI and 6 males with −9.3% (*p* = 0.09, *t*-test), which is consistent with the above proposal. In this regard, Kumari et al. (2010) reported that progesterone plays a role in balancing facilitation/inhibition. In the present study, the majority of female subjects with PPF were younger than 50 years of age (Figure 3D), suggesting that this mechanism contributed to weaker PPI in females.

Sex differences have been reported in the brain that are independent of gonadal hormone levels (Ingalhalikar et al., 2014; Pandya et al., 2019; Gegenhuber and Tollkuhn, 2020; Williams et al., 2021). Among them, differences in inhibitory synaptic transmission appear to be important (Uhl et al., 2022). The excitability of a neural circuit is controlled by excitatory pyramidal neurons and inhibitory interneurons. Since gamma-amino butyric acid (GABA) is the main inhibitory neurotransmitter in the brain, GABAergic transmission is expected to be involved in PPS and PPI (Kodsi and Swerdlow, 1995; Fendt et al., 2001; Inui et al., 2018). In a study using post-mortem brain tissues and Western Blotting, Pandya et al. (2019) found that in the superior temporal gyrus (STG), the neural source of the present auditory change-related response (Inui et al., 2010b, 2012), GABA-A receptor expression was markedly higher in males than in females; the α1 subunit in both the young and older age groups and the α2, α5, and β3 subunits in the older age groups, which is consistent with the present results showing stronger inhibition in males (Table 1). This sex bias and stronger test- or onset-evoked responses in females in the present study is consistent given that excitation and inhibition always coexist in the neural circuit of the change-related response (Inui et al., 2016).

The findings of previous studies using AEPs are inconsistent. The amplitude of AEPs was greater in females for N100 (Xin et al., 2021), but greater in males for P50 (Fuerst et al., 2007) and N100 (Fuerst et al., 2007; Golob et al., 2007), while no differences were observed for P50 (Hetrick et al., 1996; Rasco et al., 2000; Clementz and Blumenfeld, 2001; Brinkman and Stauder, 2007; Golob et al., 2007; Lijffijt et al., 2009a; Xin et al., 2021), N100 (Hetrick et al., 1996; Clementz and Blumenfeld, 2001; Lijffijt et al., 2009a), or P200 (Hetrick et al., 1996; Lijffijt et al., 2009a; Xin et al., 2021). Sex differences in PPS are also inconsistent. While some studies reported the stronger inhibition of P50 or N100 for males (Hetrick et al., 1996; Fuerst et al., 2007), others demonstrated the opposite (Xin et al., 2021) or no differences (Rasco et al., 2000; Clementz and Blumenfeld, 2001; Brinkman and Stauder, 2007; Lijffijt et al., 2009a). In the present study, the amplitudes of both the change-related test response (S1 and Test alone) and onset response were greater in females than in males. Since the onset response is a type of change-related response against the silent background (Nishihara et al., 2011), these results suggest that the cortical response to auditory changes is stronger in females. The change-related cortical response is a subtype of defense reactions (Inui et al., 2012) similar to startle reflexes (Turpin, 1986); therefore, stronger change-related responses in females are consistent with the flexion reflex (Sandrini et al., 2005), auditory startle reflex (Kofler et al., 2001), and PPF of the startle reflex (Aasen et al., 2005) being stronger in females. This information is important for understanding clinical conditions that show sex differences. For example, females are vulnerable to stress-induced anxiety, with the prevalence of panic disorder being two- to three-fold higher than in males (Donner and Lowry, 2013). In the present study, females responded more strongly to subtle sensory changes than males, but were less able to inhibit the process, which may lead to excessive automatic responses to the ever-changing environment in females. A previous study demonstrated that the amplitude of the change-related cortical response correlated with trait anxiety (Tanahashi et al., 2016).

### 4.2 Effects of age

The present results showed that aging had a significant effect on the S1 amplitude, onset amplitude, and PPS. The increase in S1 and decrease in PPS indicate a decrease in inhibitory ability with age and a consequential increase in the S1 amplitude, which is consistent with previous AEP studies showing greater amplitudes in older than in younger subjects (Judd et al., 1992; Amenedo and Díaz, 1998; Bennett et al., 2004; Golob et al., 2007; Sörös et al., 2009) and less somatosensory gating in older subjects (Lenz et al., 2012; Terrasa et al., 2018). Although the mechanisms underlying reduced inhibition in the elderly remain unclear, a reduction in GABAergic neurons in the thalamus has been proposed (Woods and Clayworth, 1986; Amenedo and Díaz, 1998). Since inhibitory interneurons are ubiquitous components of all neural circuits (Isaacson and Scanziani, 2011), the auditory cortex is also a candidate site for age-related modulation. In this regard, a study using post-mortem brain tissues showed that glutamic acid decarboxylase expression, an enzyme that synthesizes GABA, and α3 subunit expression in STG were higher in younger than in older subjects (Pandya et al., 2019). Although the GABA receptor subtype involved in PPS and PPI in the present study was unclear, the results of Exp1 are consistent with these findings. Therefore, these findings support the inhibition deficit hypothesis of cognitive aging (Hasher and Zacks, 1988), in which decreased inhibition in the elderly results in impairments in various cognitive aspects, such as concentration and working memory. In a recent study, Alain et al. (2022) claimed that enhanced neural activity in the elderly was due to the deterioration of supra-modal brain areas. They recorded sensory evoked potentials in the somatosensory, visual, and auditory systems and found that older subjects showed significantly stronger responses in all modalities. In addition, older subjects who showed enhanced neural activity in one sensory system also exhibited enhanced activity in other systems, leading to the conclusion of a common cause of age-related decline in sensory systems. Therefore, in addition to the sensory cortex, brain areas outside of it, e.g., the prefrontal cortex, are a potential site of modulation.

An important result in the present study was that aging was resistant to PPI in Exp2, which is inconsistent with the generalized decline in inhibitory function with aging. Some cognitive processes are considered to be less susceptible to aging, e.g., automatic processes (Hasher and Zacks, 1979), and the inhibition of PPI noted in this study may be involved in these functions. The change-related cortical response used in the present study as the test response is automatic and elicited without subjects’ attention (Inui et al., 2010a) and is considered to be a fundamental function for survival (Inui et al., 2012). Although there are some paradigms for observing PPI, each PPI appears to reflect a similar automatic process (Ellwanger et al., 2003). A recent study using the trigeminal blink reflex showed that PPI of the early component of the blink reflex occurred in the first stage of brain processing in the principal nucleus of the trigeminal nerve and did not involve higher complex processes (Inui et al., 2023). In studies with PPI of the acoustic startle reflex, one reported decreased inhibition in older subjects (de Oliveira et al., 2023), while others reported no effect of age (Filion and Poje, 2003; Scholes and Martin-Iverson, 2009) or an inverted U-shaped relationship between age and PPI, with the strongest inhibition in middle age (Ellwanger et al., 2003). PPI of the trigeminal blink reflex by somatosensory inputs showed no age effect (Kofler et al., 2013). These findings are generally in line with the present results and support the view that whatever the paradigm, PPI reflects age-resistant inhibitory function. In this regard, the finding reported by Ueki et al. (2006) is important: PPI of the acoustic startle reflex was reduced in patients with Alzheimer’s disease, but enhanced in patients with mild cognitive impairments, which suggests that the measure differentiates abnormal cognitive impairment from normal aging and pathophysiological changes in the early stage from advanced stages.

### 4.3 Methodological consideration

The PPI of the acoustic startle reflex and the current PPI have similar age and sex biases, but different age and sex effects on the test response: the elderly showed a weaker acoustic startle reflex in previous studies (Ellwanger et al., 2003; de Oliveira et al., 2023), but not in the present study (Figure 3C), and females showed stronger test alone responses in this study, but not for the acoustic startle reflex (Kumari et al., 2004). The most important methodological difference may be that the acoustic startle reflex was the peripheral response in previous studies and the brain response in the present study. These findings indicate that muscle activity, but not the change-related response is affected by aging, while sex has an impact on the change-related response, but not muscle activity. These variables need to be considered when designing a study.

Regarding the effect of age on the magnitude of the test response, the weaker acoustic startle reflex and its decreased inhibition by the prepulse is problematic because both may be attributed to a reduction in peripheral activity. On the other hand, in Exp1 in the present study, age correlated with a stronger test response and decreased PPS, suggesting that a decline in the periphery was not the cause of decreased inhibition. However, the underlying mechanisms appear to be complex: in older individuals, reduced afferent inputs to the brain result in the loss of GABAergic inhibition, which, in turn, enhances brain activity (Harris et al., 2022). Therefore, the results obtained in Exp1 are in agreement with the central gain hypothesis (Gutiérrez et al., 1994; Turrigiano and Nelson, 2004). The weaker acoustic startle reflex in older subjects may be due to an age-related decline in the trigeminal blink system. This needs to be confirmed in further studies with older subjects and larger sample sizes than those in the present study.

## 5 Limitations

A limitation of the present study is that although the results obtained clearly showed sex effects in both measures, its significance has yet to be clarified due to the lack of information on menstruation or the contraceptive status in the present study. Another limitation is the component of evoked potentials tested. Previous studies reported differences in age and sex effects among components. In the present study, the peak-to-peak amplitude was employed to simplify the results, leaving the possibility that distinct effects for each component were not examined. As shown in Supplementary Tables S1, S2, there were some minor differences among components; however, it is difficult to establish whether these differences are meaningful. Large variations in P50 are problematic, with some data showing negative values. This is largely due to the filter setting in the present study; higher low-cut filters are necessary for P50, but are not appropriate for later components. We consider the peak-to-peak amplitude to be a good method due to the lack of baseline shift issues and the high signal-to-noise ratio.

## 6 Conclusion

The effects of age and sex on PPS and PPI of auditory change-related cortical responses were investigated. Sex showed a similar bias in both tests with stronger test responses and weaker inhibition in females. Age affected the two measures differently, with the test response being stronger and inhibition being weaker in older subjects in the PPS paradigm, while no age effects were observed in the test alone response amplitude or the degree of inhibition in the PPI paradigm. Although the neural mechanisms of PPS and PPI in humans currently remain unclear, some of the mechanisms proposed for PPS, such as the depletion of transmitters, are unlikely for PPI. In addition, the most effective interval of the two successive stimuli differs between PPS and PPI; 600 ms for PPS (Takeuchi et al., 2017) and 20–60 ms for PPI (Inui et al., 2016). The inhibition threshold also differs (Inui et al., 2016). Therefore, the two methods represent distinct inhibitory mechanisms. The present results showing the different impact of age on PPS and PPI and the lack of a correlation between the degree of PPS and PPI support this notion. In comparisons with studies using different paradigms, some of the findings obtained were congruent with the present results, whereas others were not. Since these measures are widely impaired in neuropsychiatric diseases (Adler et al., 1982; Jessen et al., 2001; Swerdlow et al., 2008; Santos-Carrasco and De la Casa, 2023), they are not of high diagnostic value. However, each method has a common and unique neural basis, which may be useful for insights into pathophysiology when considered in combination.

## Data availability statement

The original contributions presented in the study are included in the article/Supplementary material, further inquiries can be directed to the corresponding author.

## Ethics statement

The studies involving humans were approved by Ethics Committee of the Aichi Developmental Disability Center, Kasugai, Japan. The studies were conducted in accordance with the local legislation and institutional requirements. The participants provided their written informed consent to participate in this study.

## Author contributions

KI: Conceptualization, Data curation, Formal analysis, Funding acquisition, Writing – original draft. NT: Data curation, Formal analysis, Methodology, Writing – review & editing. BB: Data curation, Formal analysis, Writing – review & editing. MS: Data curation, Formal analysis, Writing – review & editing. SS: Data curation, Formal analysis, Supervision, Writing – review & editing. TT: Data curation, Formal analysis, Writing – review & editing. MN: Data curation, Formal analysis, Supervision, Writing – review & editing. TW: Data curation, Formal analysis, Writing – review & editing. DS: Data curation, Formal analysis, Writing – review & editing. EM: Conceptualization, Data curation, Formal analysis, Supervision, Writing – review & editing. TK: Data curation, Formal analysis, Software, Supervision, Writing – review & editing.
