# Supplementary material for: Age and sex effects on paired-pulse suppression and prepulse inhibition of auditory evoked potentials
Source: Front Neurosci. 2024 Apr 9;18:1378619. doi: 10.3389/fnins.2024.1378619 (PMC11035799; doi:10.3389/fnins.2024.1378619)
Supplement: Supplementary file 1 [file Table_1.DOCX]

Supplementary Material

# Supplementary Table 1. Differences in each evoked potential component between sexes.

The results of *t*-tests using peak-to-peak amplitudes (P50/N100 and N100/P200) and each component amplitude are shown. Some data with negative P50 and positive N100 in the test response were excluded from the analysis in this and subsequent tables because of the difficulties associated with calculating the inhibition rate. In the peak-to-peak amplitude, this was not an issue.


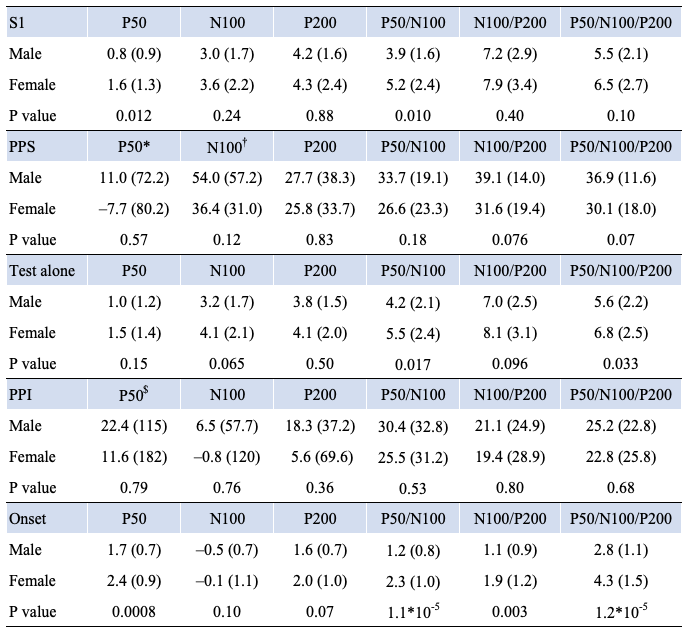


Data are shown as means (SD). S1, Test alone, and Onset indicate the amplitude in µV, while PPS and PPI indicate the inhibition rate in %.

* n=58 (32 females and 26 males) who showed the S1 response with positive P50.

† n=66 (34 females and 32 males) who showed the S1 response with negative N100.

$ n=59 (32 females and 27 males) who showed the test alone response with positive P50.

**Supplementary Table 2. Correlations between age and five variables using each evoked potential component.**

**
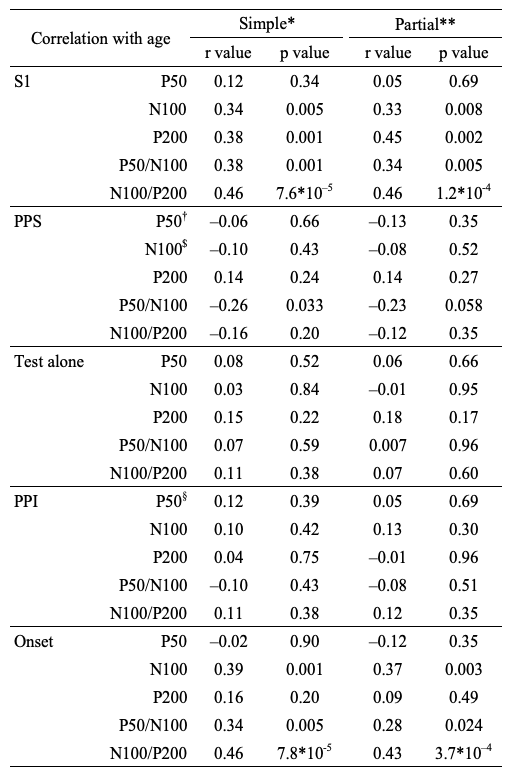
**

* Simple linear correlation coefficients (Pearson)

** Partial correlation coefficients controlling sex

† n=58 (32 females and 26 males) who showed the S1 response with positive P50.

$ n=66 (34 females and 32 males) who showed the S1 response with negative N100.

§ n=59 (32 females and 27 males) who showed the test alone response with positive P50
